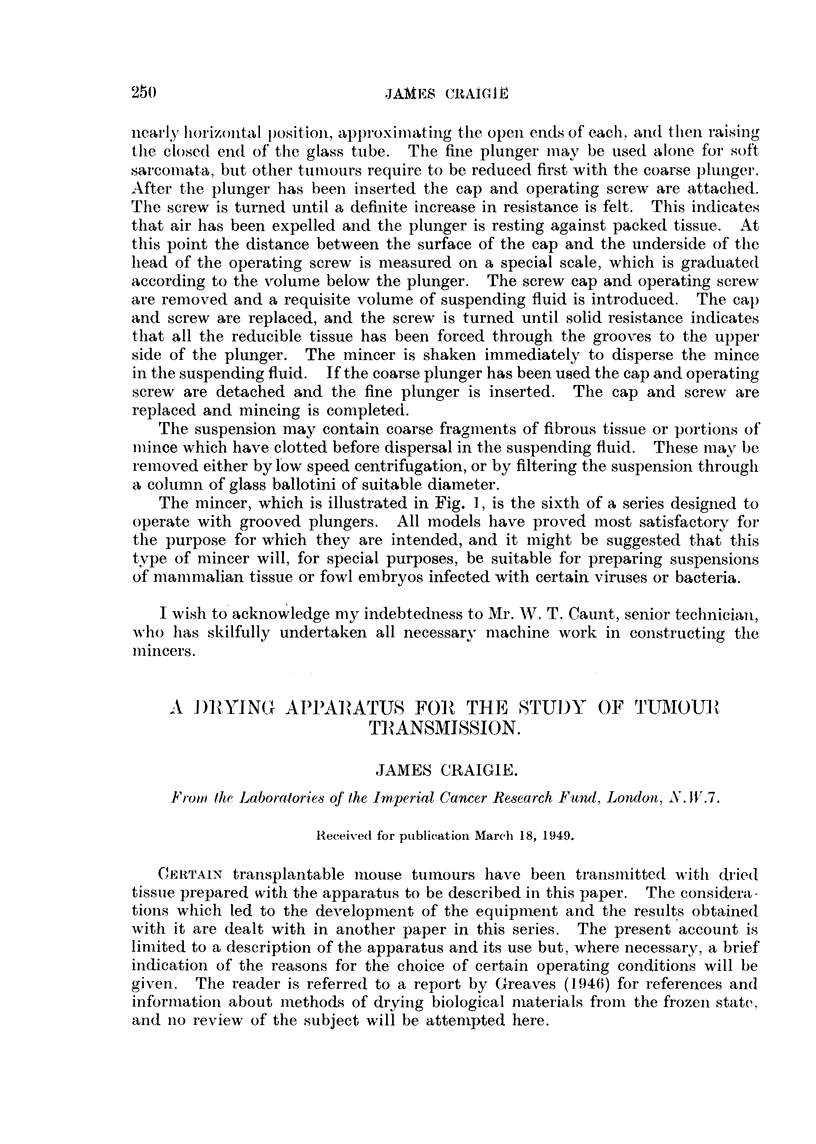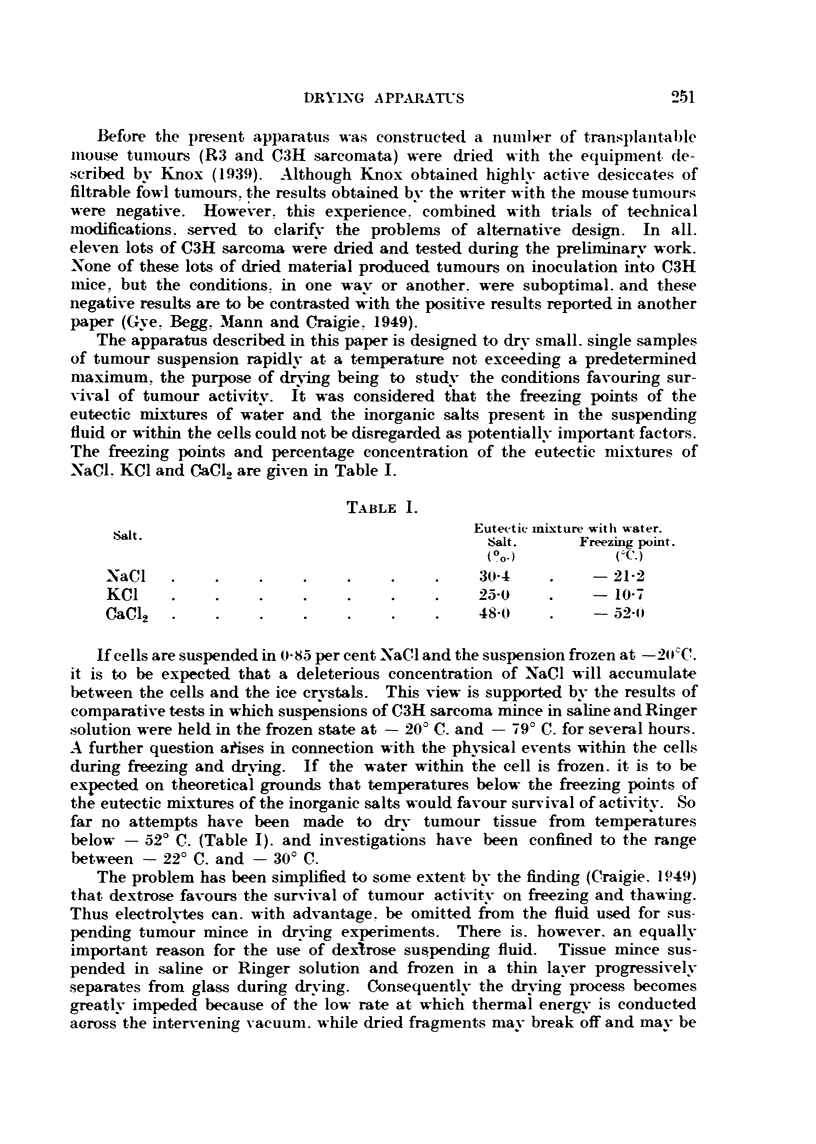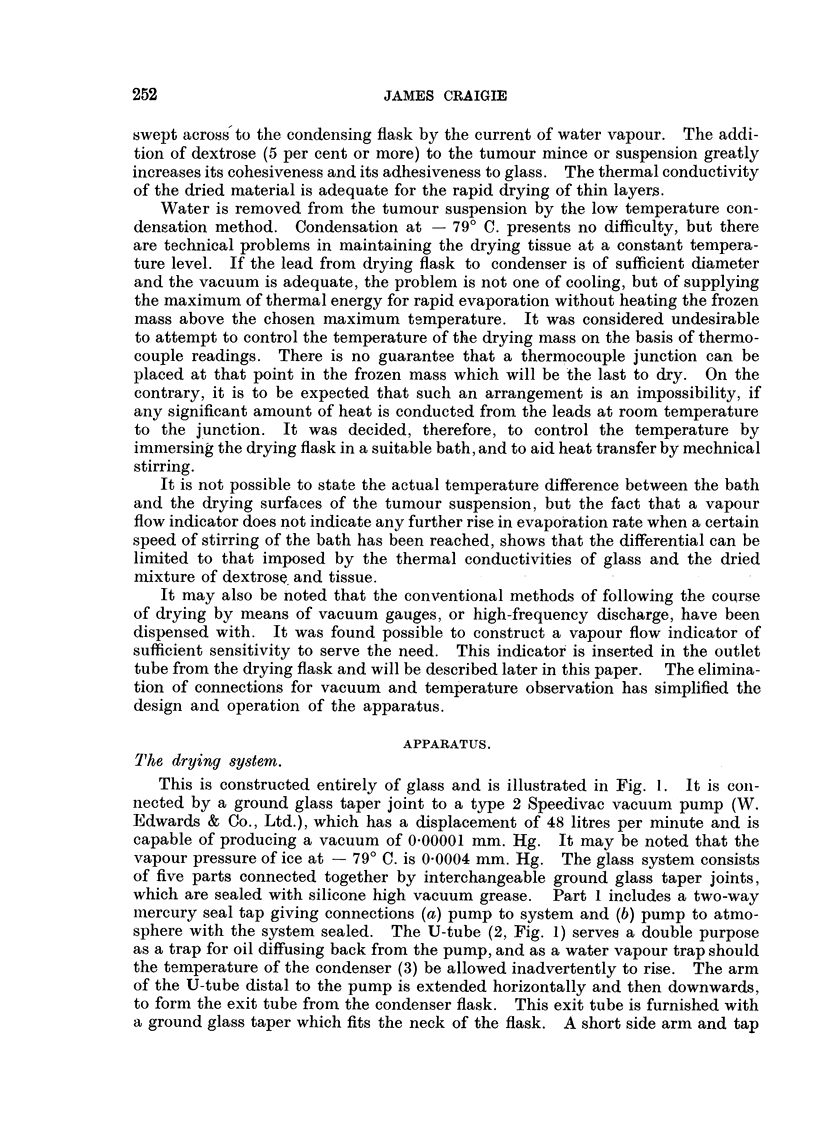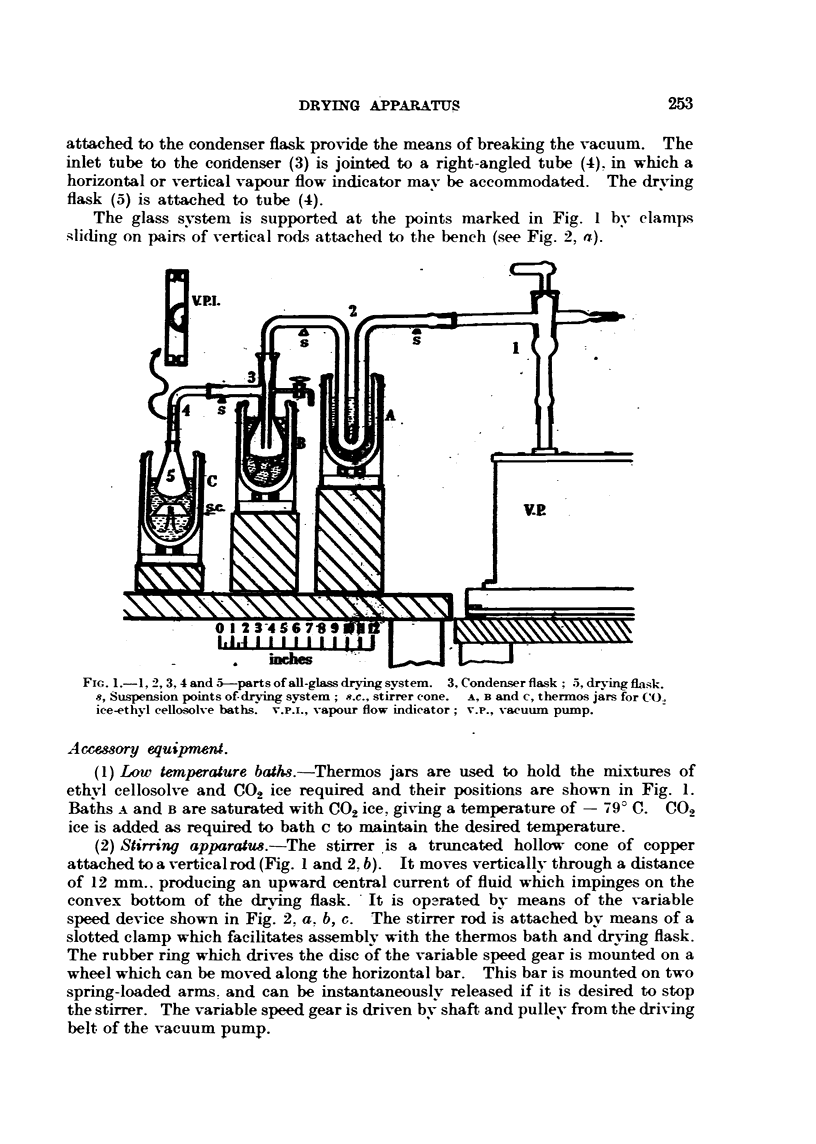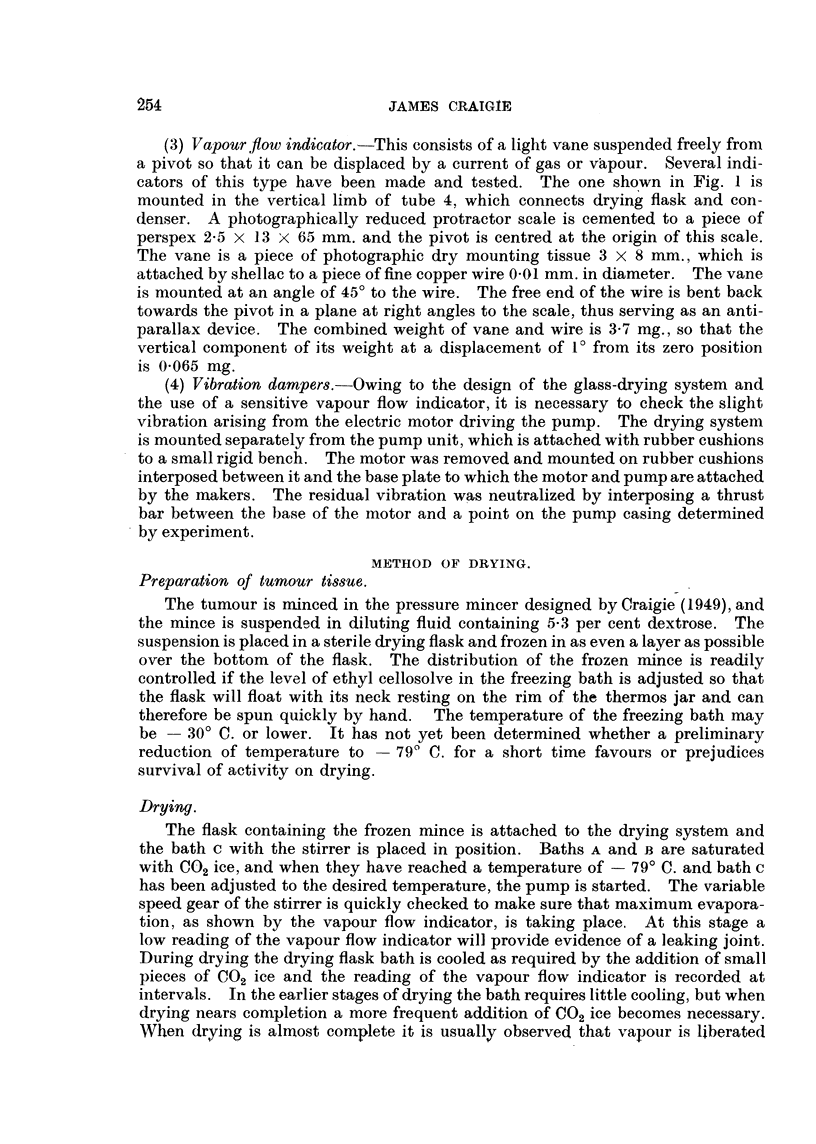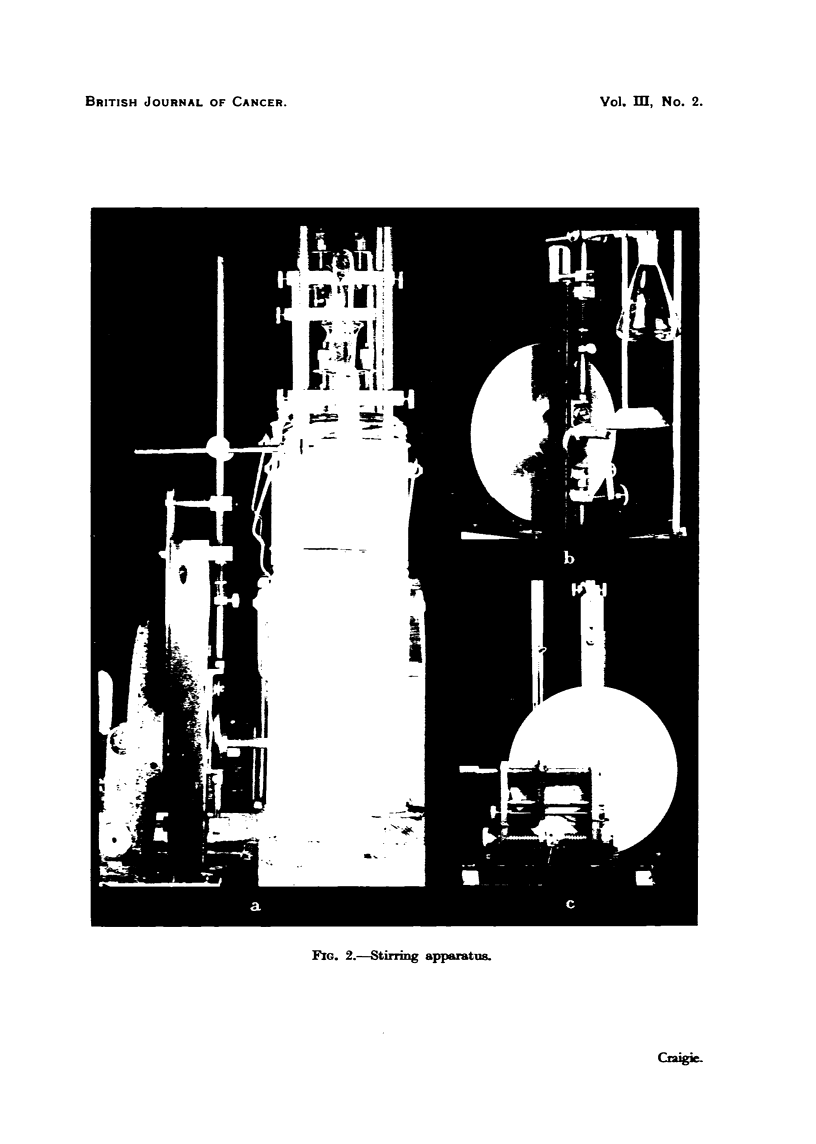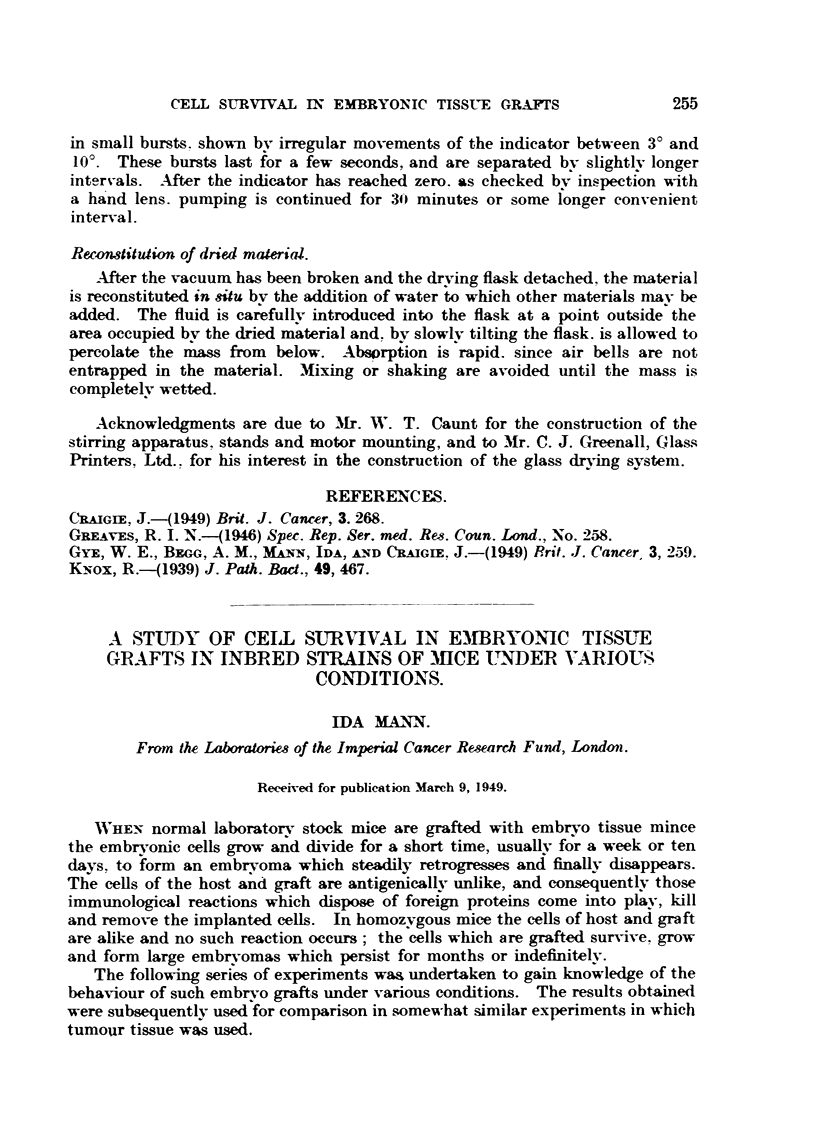# A Drying Apparatus for the Study of Tumour Transmission

**DOI:** 10.1038/bjc.1949.27

**Published:** 1949-06

**Authors:** James Craigie

## Abstract

**Images:**


### A DRYING APPARATUS FOR THE STUDY OF TUMOUR TRANSMISSION.

JAMES CRAIGIE.

*From the Laboratories of the Imperial Cancer Research Fund, London, N.W.7.*

Received for publication March 18, 1949.

CERTAIN transplantable mouse tumours have been transmitted with dried tissue prepared with the apparatus to be described in this paper. The considerations which led to the development of the equipment and the results obtained with it are dealt with in another paper in this series. The present account is limited to a description of the apparatus and its use but, where necessary, a brief indication of the reasons for the choice of certain operating conditions will be given. The reader is referred to a report by Greaves (1946) for references and information about methods of drying biological materials from the frozen state, and no review of the subject will be attempted here.

Before the present apparatus was constructed a number of transplantable mouse tumours (R3 and C3H sarcomata) were dried with the equipment described by Knox (1939). Although Knox obtained highly active desiccates of filtrable fowl tumours, the results obtained by the writer with the mouse tumours were negative. However, this experience, combined with trials of technical modifications, served to clarify the problems of alternative design. In all, eleven lots of C3H sarcoma were dried and tested during the preliminary work. None of these lots of dried material produced tumours on inoculation into C3H mice, but the conditions, in one way or another, were suboptimal, and these negative results are to be contrasted with the positive results reported in another paper (Gye, Begg, Mann and Craigie, 1949).

The apparatus described in this paper is designed to dry small, single samples of tumour suspension rapidly at a temperature not exceeding a predetermined maximum, the purpose of drying being to study the conditions favouring survival of tumour activity. It was considered that the freezing points of the eutectic mixtures of water and the inorganic salts present in the suspending fluid or within the cells could not be disregarded as potentially important factors. The freezing points and percentage concentration of the eutectic mixtures of NaCl, KCl and $CaCl_2$ are given in Table I.

TABLE I.

| Salt. | Eutectic mixture with water. Salt. (%.) | Freezing point. (°C.) |
|---|---|---|
| NaCl | 30·4 | — 21·2 |
| KCl | 25·0 | — 10·7 |
| $CaCl_2$ | 48·0 | — 52·0 |

If cells are suspended in 0·85 per cent NaCl and the suspension frozen at —20°C. it is to be expected that a deleterious concentration of NaCl will accumulate between the cells and the ice crystals. This view is supported by the results of comparative tests in which suspensions of C3H sarcoma mince in saline and Ringer solution were held in the frozen state at — 20° C. and — 79° C. for several hours. A further question arises in connection with the physical events within the cells during freezing and drying. If the water within the cell is frozen, it is to be expected on theoretical grounds that temperatures below the freezing points of the eutectic mixtures of the inorganic salts would favour survival of activity. So far no attempts have been made to dry tumour tissue from temperatures below — 52° C. (Table I), and investigations have been confined to the range between — 22° C. and — 30° C.

The problem has been simplified to some extent by the finding (Craigie, 1949) that dextrose favours the survival of tumour activity on freezing and thawing. Thus electrolytes can, with advantage, be omitted from the fluid used for suspending tumour mince in drying experiments. There is, however, an equally important reason for the use of dextrose suspending fluid. Tissue mince suspended in saline or Ringer solution and frozen in a thin layer progressively separates from glass during drying. Consequently the drying process becomes greatly impeded because of the low rate at which thermal energy is conducted across the intervening vacuum, while dried fragments may break off and may be swept across to the condensing flask by the current of water vapour. The addition of dextrose (5 per cent or more) to the tumour mince or suspension greatly increases its cohesiveness and its adhesiveness to glass. The thermal conductivity of the dried material is adequate for the rapid drying of thin layers.

Water is removed from the tumour suspension by the low temperature condensation method. Condensation at − 79° C. presents no difficulty, but there are technical problems in maintaining the drying tissue at a constant temperature level. If the lead from drying flask to condenser is of sufficient diameter and the vacuum is adequate, the problem is not one of cooling, but of supplying the maximum of thermal energy for rapid evaporation without heating the frozen mass above the chosen maximum temperature. It was considered undesirable to attempt to control the temperature of the drying mass on the basis of thermocouple readings. There is no guarantee that a thermocouple junction can be placed at that point in the frozen mass which will be the last to dry. On the contrary, it is to be expected that such an arrangement is an impossibility, if any significant amount of heat is conducted from the leads at room temperature to the junction. It was decided, therefore, to control the temperature by immersing the drying flask in a suitable bath, and to aid heat transfer by mechnical stirring.

It is not possible to state the actual temperature difference between the bath and the drying surfaces of the tumour suspension, but the fact that a vapour flow indicator does not indicate any further rise in evaporation rate when a certain speed of stirring of the bath has been reached, shows that the differential can be limited to that imposed by the thermal conductivities of glass and the dried mixture of dextrose and tissue.

It may also be noted that the conventional methods of following the course of drying by means of vacuum gauges, or high-frequency discharge, have been dispensed with. It was found possible to construct a vapour flow indicator of sufficient sensitivity to serve the need. This indicator is inserted in the outlet tube from the drying flask and will be described later in this paper. The elimination of connections for vacuum and temperature observation has simplified the design and operation of the apparatus.

### APPARATUS.

### *The drying system.*

This is constructed entirely of glass and is illustrated in Fig. 1. It is connected by a ground glass taper joint to a type 2 Speedivac vacuum pump (W. Edwards & Co., Ltd.), which has a displacement of 48 litres per minute and is capable of producing a vacuum of 0·00001 mm. Hg. It may be noted that the vapour pressure of ice at − 79° C. is 0·0004 mm. Hg. The glass system consists of five parts connected together by interchangeable ground glass taper joints, which are sealed with silicone high vacuum grease. Part 1 includes a two-way mercury seal tap giving connections (*a*) pump to system and (*b*) pump to atmosphere with the system sealed. The U-tube (2, Fig. 1) serves a double purpose as a trap for oil diffusing back from the pump, and as a water vapour trap should the temperature of the condenser (3) be allowed inadvertently to rise. The arm of the U-tube distal to the pump is extended horizontally and then downwards, to form the exit tube from the condenser flask. This exit tube is furnished with a ground glass taper which fits the neck of the flask. A short side arm and tap attached to the condenser flask provide the means of breaking the vacuum. The inlet tube to the condenser (3) is jointed to a right-angled tube (4), in which a horizontal or vertical vapour flow indicator may be accommodated. The drying flask (5) is attached to tube (4).

The glass system is supported at the points marked in Fig. 1 by clamps sliding on pairs of vertical rods attached to the bench (see Fig. 2, *a*).

FIG. 1.—1, 2, 3, 4 and 5—parts of all-glass drying system. 3, Condenser flask ; 5, drying flask. *s*, Suspension points of drying system ; *s.c.*, stirrer cone. A, B and C, thermos jars for $CO_2$ ice-ethyl cellosolve baths. V.P.I., vapour flow indicator ; V.P., vacuum pump.

*Accessory equipment.*

(1) *Low temperature baths.*—Thermos jars are used to hold the mixtures of ethyl cellosolve and $CO_2$ ice required and their positions are shown in Fig. 1. Baths A and B are saturated with $CO_2$ ice, giving a temperature of $- 79°$ C. $CO_2$ ice is added as required to bath C to maintain the desired temperature.

(2) *Stirring apparatus.*—The stirrer is a truncated hollow cone of copper attached to a vertical rod (Fig. 1 and 2, *b*). It moves vertically through a distance of 12 mm., producing an upward central current of fluid which impinges on the convex bottom of the drying flask. It is operated by means of the variable speed device shown in Fig. 2, *a*, *b*, *c*. The stirrer rod is attached by means of a slotted clamp which facilitates assembly with the thermos bath and drying flask. The rubber ring which drives the disc of the variable speed gear is mounted on a wheel which can be moved along the horizontal bar. This bar is mounted on two spring-loaded arms, and can be instantaneously released if it is desired to stop the stirrer. The variable speed gear is driven by shaft and pulley from the driving belt of the vacuum pump.

(3) *Vapour flow indicator.*—This consists of a light vane suspended freely from a pivot so that it can be displaced by a current of gas or vapour. Several indicators of this type have been made and tested. The one shown in Fig. 1 is mounted in the vertical limb of tube 4, which connects drying flask and condenser. A photographically reduced protractor scale is cemented to a piece of perspex 2·5 × 13 × 65 mm. and the pivot is centred at the origin of this scale. The vane is a piece of photographic dry mounting tissue 3 × 8 mm., which is attached by shellac to a piece of fine copper wire 0·01 mm. in diameter. The vane is mounted at an angle of 45° to the wire. The free end of the wire is bent back towards the pivot in a plane at right angles to the scale, thus serving as an anti-parallax device. The combined weight of vane and wire is 3·7 mg., so that the vertical component of its weight at a displacement of 1° from its zero position is 0·065 mg.

(4) *Vibration dampers.*—Owing to the design of the glass-drying system and the use of a sensitive vapour flow indicator, it is necessary to check the slight vibration arising from the electric motor driving the pump. The drying system is mounted separately from the pump unit, which is attached with rubber cushions to a small rigid bench. The motor was removed and mounted on rubber cushions interposed between it and the base plate to which the motor and pump are attached by the makers. The residual vibration was neutralized by interposing a thrust bar between the base of the motor and a point on the pump casing determined by experiment.

### METHOD OF DRYING.

### *Preparation of tumour tissue.*

The tumour is minced in the pressure mincer designed by Craigie (1949), and the mince is suspended in diluting fluid containing 5·3 per cent dextrose. The suspension is placed in a sterile drying flask and frozen in as even a layer as possible over the bottom of the flask. The distribution of the frozen mince is readily controlled if the level of ethyl cellosolve in the freezing bath is adjusted so that the flask will float with its neck resting on the rim of the thermos jar and can therefore be spun quickly by hand. The temperature of the freezing bath may be − 30° C. or lower. It has not yet been determined whether a preliminary reduction of temperature to − 79° C. for a short time favours or prejudices survival of activity on drying.

### *Drying.*

The flask containing the frozen mince is attached to the drying system and the bath C with the stirrer is placed in position. Baths A and B are saturated with $CO_2$ ice, and when they have reached a temperature of − 79° C. and bath C has been adjusted to the desired temperature, the pump is started. The variable speed gear of the stirrer is quickly checked to make sure that maximum evaporation, as shown by the vapour flow indicator, is taking place. At this stage a low reading of the vapour flow indicator will provide evidence of a leaking joint. During drying the drying flask bath is cooled as required by the addition of small pieces of $CO_2$ ice and the reading of the vapour flow indicator is recorded at intervals. In the earlier stages of drying the bath requires little cooling, but when drying nears completion a more frequent addition of $CO_2$ ice becomes necessary. When drying is almost complete it is usually observed that vapour is liberated

FIG. 2.—Stirring apparatus.

in small bursts, shown by irregular movements of the indicator between 3° and 10°. These bursts last for a few seconds, and are separated by slightly longer intervals. After the indicator has reached zero, as checked by inspection with a hand lens, pumping is continued for 30 minutes or some longer convenient interval.

*Reconstitution of dried material.*

After the vacuum has been broken and the drying flask detached, the material is reconstituted *in situ* by the addition of water to which other materials may be added. The fluid is carefully introduced into the flask at a point outside the area occupied by the dried material and, by slowly tilting the flask, is allowed to percolate the mass from below. Absorption is rapid, since air bells are not entrapped in the material. Mixing or shaking are avoided until the mass is completely wetted.

Acknowledgments are due to Mr. W. T. Caunt for the construction of the stirring apparatus, stands and motor mounting, and to Mr. C. J. Greenall, Glass Printers, Ltd., for his interest in the construction of the glass drying system.

### REFERENCES.

Craigie, J.—(1949) *Brit. J. Cancer*, **3**. 268.

Greaves, R. I. N.—(1946) *Spec. Rep. Ser. med. Res. Coun. Lond.*, No. 258.

Gye, W. E., Begg, A. M., Mann, Ida, and Craigie, J.—(1949) *Brit. J. Cancer*, **3**, 259.

Knox, R.—(1939) *J. Path. Bact.*, **49**, 467.